# A scoring system to predict the occurrence of very late stent thrombosis following percutaneous coronary intervention for acute coronary syndrome

**DOI:** 10.1038/s41598-020-63455-0

**Published:** 2020-04-14

**Authors:** Xiang Wang, Xinxin Chen, Tao Tian, Hongzhao You, Yulin Li, Muli Wu, Xiaoyu Du, He Cai, Yang Zheng, Jie Du

**Affiliations:** 1 Department of Cardiovascular Disease Center, First Hospital of Jilin University, Jilin University, Jilin, China; 2 State Key Laboratory of Cardiovascular Disease, Cardiovascular Institute, Fuwai Hospital and National Centre for Cardiovascular Diseases, Chinese academy of Medical Sciences & Peking Union Medical College, Beijing, China; 3 Beijing Institute of Heart, Lung and Blood Vessel Diseases, Beijing Anzhen Hospital, Capital Medical University, Beijing, China; 4 Department of Cardiovascular Center, First Affiliated Hospital of Shantou University Medical College, Shantou University, Guangdong, China

**Keywords:** Cardiology, Diseases

## Abstract

We aimed to derive and validate an effective risk score to identify high-risk patients of very late stent thrombosis (VLST), following percutaneous coronary intervention (PCI) for acute coronary syndrome (ACS). Stepwise multivariable Cox regression was used to build the risk model using data from 5,185 consecutive ACS patients treated with PCI (derivation cohort) and 2,058 patients from the external validation cohort. Eight variables were independently associated with the development of VLST: history of diabetes mellitus, previous PCI, acute myocardial infarction as admitting diagnosis, estimated glomerular filtration rate <90 ml/min/1.73 m^2^, three-vessel disease, number of stents per lesion, sirolimus-eluting stent, and no post-dilation. Based on the derived score, patients were classified into low- (≤7), intermediate- (8–9), and high- (≥10) risk categories. Observed VLST rates were 0.5%, 2.2%, and 8.7% and 0.45%, 2.3%, and 9.3% across the 3 risk categories in the derivation and validation cohorts, respectively. High discrimination (c-statistic = 0.80 and 0.82 in the derivation and validation cohorts, respectively) and excellent calibration were observed in both cohorts. VLST risk score, a readily useable and efficient tool to identify high-risk patients of VLST after PCI for ACS, may aid in risk-stratification and pre-emptive decision-making.

## Introduction

Percutaneous coronary intervention (PCI) is currently the primary treatment for acute coronary syndrome (ACS), which is one of the most serious threats to human health worldwide. Very late stent thrombosis (VLST), defined as thrombosis that occurs more than 1 year after stent implantation, is a catastrophic complication of PCI and accounts for 20% of new cases of myocardial infarction (MI) after index PCI^1^. The adjusted mortality is 4-fold higher for VLST than that for MI, which is not related to a previously stented site^1^. Tens of millions of patients worldwide have received stent implants in the past decade, and registry reports show evidence of increased VLST risk up to 5 years postoperatively, with no clear evidence of attenuation despite stent iteration and recent progress in PCI technology^2^. Therefore, early identification of high-risk patients with VLST is of great importance as this could inform tailored management regiens, including intensive monitoring and intervention, which may improve patient outcomes. However, owing to the existence of confounding factors, traditional single-factor risk analysis cannot adequately identify high-risk patients at an individual level. In 2012, Dangas *et al*. developed a risk-score system for late stent thrombosis (the LST risk score), and this proved useful in predicting the development of VLST. However, the predictive ability of the score is limited (c-statistic = 0.66)^3^. There is currently no verified clinical risk score for the prediction of VLST.

In the present study, we aimed to develop a risk score for VLST based on clinical variables from a large cohort of consecutive patients with ACS and independently validate the system in another cohort with the aim of identifying patients at high risk of VLST.

## Results

### Patient characteristics

A total of 6,038 patients were eligible for the derivation cohort; of which, 236 were excluded owing to death prior to discharge, 413 subjects refused to participate in follow-up interviews, and 204 were lost to follow-up. Finally, 5,185 patients were enrolled (average age 59.85 ± 9.95 years, 68.60% male). The median length of follow-up was 1,502 days.

For the validation cohort, of the 2,438 patients identified as eligible, we excluded 88 patients who died prior to discharge, 186 who did not agree to participate in follow-up interviews, and 106 who were lost to follow-up. Finally, 2,058 patients were enrolled (average age 60.00 ± 10.22 years, 68.71% male). The median length of follow-up was 1,422 days. Supplementary Table S1 provides a comparison of the baseline and procedural characteristics as well as laboratory data between patients within the two cohorts.

The median time from index PCI to the occurrence of VLST was 697 and 803 days for the derivation and validation cohorts, respectively. The incidence of VLST was not significantly different between the derivation and validation cohorts at 1.77% and 1.70%, respectively (P = 0.829). Table 1 provides a comparison of the baseline and procedural characteristics as well as laboratory data between patients with and without VLST in the derivation cohort. A significantly larger number of patients were admitted with an acute MI among those with VLST (P < 0.001), and the peak troponin I level was higher among these patients; although, the difference was not statistically significant (P = 0.051). Patients with VLST experienced significantly more Thrombolysis in Myocardial Infarction (TIMI) flow grade 0 before PCI (P = 0.017), stent overlap was significantly higher (P = 0.013), and stent length significantly lon ger (P = 0.001) during PCI than that among subjects without VLST. There was no difference between the occurrence of slow flow (P = 0.619) and no reflow (P = 0.547) after PCI between patients with VLST and those without. More patients without VLST received dual-antiplatelet therapy (DAPT) during follow-up compar ed to those that received DAPT with VLST; although, this was not significantly different (44.37% vs. 38.04%, respectively, P = 0.226).

**Table 1:** Baseline, procedural characteristics, and laboratory test results of patients with and without VLST in the derivation cohort.

Variables	Patients without VLST (n = 5093)	Patients with VLST (n = 92)	p-value
Age (years)	59.83 ± 9.93	60.71 ± 11.33	0.211
Male sex	3489(68.51)	68(73.91)	0.268
Diabetes Mellitus	1293(25.39)	36(39.13)	0.003
Hypertension	2670(52.42)	51(55.43)	0.567
Dyslipidaemia	1612(31.65)	28(30.43)	0.804
Current smoker	2860(56.16)	54(58.70)	0.626
History of drinking	751(14.75)	13(14.13)	0.869
History of stroke/TIA	367(7.21)	12(13.04)	0.033
History of PVD	86(1.69)	2(2.17)	0.670
History of heart failure	135(2.65)	2(2.17)	0.559
Previous CABG	140(2.75)	4(4.35)	0.325
Previous PCI	94(1.85)	13(14.13)	<0.001
Previous MI	347(6.81)	10(10.87)	0.128
AMI as admitting diagnosis	3046(59.81)	72(78.26)	<0.001
Cardiogenic shock	110(2.16)	3(3.26)	0.454
Three-vessel disease	1422(27.92)	42(45.65)	<0.001
Chronic total occlusion	235(4.61)	4(4.35)	0.580
Moderate to severe calcification	182(3.57)	3(3.26)	0.583
Moderate to severe tortuosity	59(1.16)	1(1.09)	0.712
Ostial lesion	117(2.30)	2(2.17)	0.646
Proximal lesion	3481(68.35)	63(68.48)	0.979
Bifurcation lesion	621(12.19)	17(18.48)	0.069
Visual thrombus	588(11.55)	10(10.87)	0.841
Coronary aneurysm	27(0.53)	1(1.09)	0.395
Vessel ectasia	57(1.11)	2(2.17)	0.282
Vessel ulceration	38(0.75)	1(1.09)	0.504
Vessel dissection	52(1.02)	2(2.17)	0.249
**Culprit vessel**			
LM	103(2.02)	2(2.17)	0.710
LAD	2682(52.66)	47(51.09)	0.764
LCX	879(17.26)	17(18.48)	0.759
RCA	1689(33.16)	32(34.78)	0.744
TIMI flow grade 0 before PCI	1668(32.75)	41(44.57)	0.017
Thrombus aspiration	654(12.84)	10(10.87)	0.575
Slow flow after PCI	173(3.40)	3(3.26)	0.619
No reflow after PCI	43(0.84)	1(1.08)	0.547
**Stent type used during PCI**			
SES	3502(68.76)	76(82.61)	0.004
ZES	889(17.46)	9(9.78)	0.054
EES	702(13.78)	7(7.61)	0.088
Stent overlap	968(19.01)	27(29.35)	0.013
No post-dilation	2496(49.01)	14(15.22)	<0.001
Reference vessel diameter (mm)	3.03 ± 0.40	2.99 ± 0.36	0.541
Min-stent diameter (mm)	2.99 ± 0.41	2.92 ± 0.39	0.117
Max-stent diameter (mm)	3.06 ± 0.41	3.05 ± 0.38	0.826
Total stent length (mm)	31.70 ± 15.62	38.46 ± 20.71	0.001
Stent release pressure (atm)	13.95 ± 2.99	13.93 ± 2.80	0.901
No. of stents per lesion	1.21 ± 0.46	1.42 ± 0.65	<0.001
LVEF	55.15 ± 4.52	54.55 ± 3.68	0.033
Peak troponin I	37.37 ± 61.64	40.55 ± 72.25	0.051
eGFR <90(ml/min/1.73 m^2^)	860(16.89)	32(34.78)	<0.001
WBC (*10^9^/l)	8.75 ± 3.24	12.49 ± 3.92	<0.001
HGB (g/l)	141.64 ± 16.22	144.53 ± 13.08	0.171
Platelet (*10^9^/l)	225.31 ± 62.49	224.24 ± 56.09	0.816
TCL (mmol/l)	4.57 ± 1.05	4.69 ± 1.31	0.756
LDL (mmol/l)	2.86 ± 0.81	3.05 ± 1.08	0.327
HDL (mmol/l)	1.13 ± 0.28	1.13 ± 0.35	0.672
TG (mmol/l)	2.12 ± 1.39	2.32 ± 1.71	0.558
Fasting blood-glucose (mmol/l)	6.70 ± 2.73	7.82 ± 3.98	0.025
HbA1c (%)	6.27 ± 1.53	5.80 ± 1.61	<0.001
Fibrinogen (g/l)	3.17 ± 0.88	3.37 ± 1.01	0.012
DAPT	2260(44.37)	35(38.04)	0.226

AMI, acute myocardial infarction; CABG, coronary artery bypass graft; DAPT, dual-antiplatelet therapy; EES, everolimus eluting stent; eGFR, estimated glomerular filtration rate; HDL, high density lipoprotein; HGB, haemoglobin; LAD, left anterior descending artery; LCX, left circumflex artery; LDL, low density lipoprotein; LM, left main; LVEF, left ventricular eject fraction; MI, myocardial infarction; PCI, percutaneous coronary intervention; PVD, peripheral vascular disease; RCA, right coronary artery; SES, sirolimus-eluting stent; TCL, total cholesterol; TG, triglyceride; TIA, transient ischemic attack; TIMI, Thrombolysis in Myocardial Infarction; VLST, very late stent thrombosis; WBC, white blood cell; ZES, zotarolimus eluting stent

### Development of the very late stent thrombosis risk score

In total, 12 predictors were identified for the development of VLST from the univariable analysis in the derivation cohort, and 4 independent risk factors were identified in the literature (Supplementary Table S2). Eight predictors were independently associated with the occurrence of VLST by multivariable Cox regression analysis as follows: history of diabetes mellitus (DM), previous PCI, acute MI as the admitting diagnosis, estimated glomerular filtration rate (eGFR) of <90 ml/min/1.73 m^2^, three-vessel disease, number of stents per lesion, sirolimus-eluting stent (SES), and no post-dilation. The variance-inflation factor was <2, indicating the absence of multicollinearity among variables in the model. The p-value from the Hosmer-Lemeshow goodness-of-fit test of the final model was 0.082 for the derivation cohort. The c-statistic of the final model was 0.81 (95% confidence interval [CI]: 0.76–0.86), and cross-validation predicted a slight decrease in discriminative ability (c-statistic 0.75, 95% CI: 0.70–0.80).

**Figure 1:**
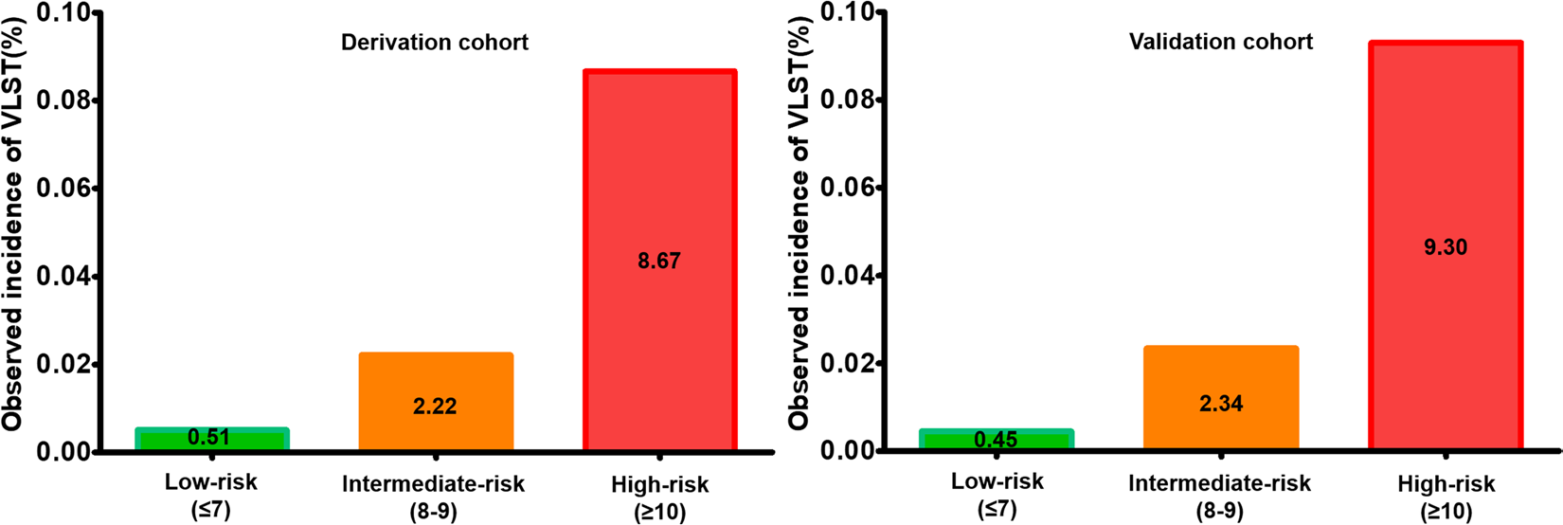
Observed incidence of VLST. Observed incidence of VLST according to the categories of the VLST risk score in the derivation and validation cohorts. VLST, very late stent thrombosis.

The points assigned to each variable of the risk-scoring system are shown in Table 2. The relationship between score value and the predicted incidence of VLST is illustrated in Supplementary Fig. S1 (P = 0.345 from the Hosmer-Lemeshow test of the score). Supplementary Fig. S2 presents the calibration plot of predicted vs. observed incidence of VLST development among the derivation cohort across deciles of the risk score, which shows excellent calibration. The c-statistic and hazard ratio (HR) of the score was 0.80 (95% CI: 0.75–0.85) and 1.69 (95% CI: 1.54–1.85), respectively, in the derivation cohort. Levels of risk were defined according to the predicted incidence of VLST: low risk = score of ≤7, corresponding to a predicted incidence of <1% (3,135 patients, 60.5% of the derivation cohort); intermediate risk = 8 or 9 points, corresponding to a predicted incidence of 1%–3% (1,837 patients, 35.4% of the derivation cohort); high-risk was ≥10 points, corresponding to a predicted incidence of >3% (473 patients, 9.1% of the derivation cohort). The observed incidences of VLST according to these cut-offs were 0.5%, 2.2%, and 8.7%, respectively, in the derivation cohort (Fig. 1).

**Table 2:** Multivariable predictors of VLST in the derivation cohort and their respective weights in the VLST risk score.

Variables	Corrected β	Multivariable HR (95% CI)	p-value	Risk score assigned weight
Diabetes Mellitus	0.58	1.78(1.17–2.72)	0.008	1
Previous PCI	1.68	5.34(2.72–10.45)	<0.001	3
AMI as admitting diagnosis	0.54	1.71(1.07–2.72)	0.024	1
eGFR<90(ml/min/1.73 m^2^)	0.76	2.13(1.38–3.29)	0.001	1
3-vessel disease	0.51	1.66(1.09–2.53)	0.019	1
No. of stents per lesion	0.96	2.62(1.90–3.61)	<0.001	2*No.
Stent type-SES	0.66	1.94(1.13–3.34)	0.017	1
No post-dilation	1.93	0.145(0.08–0.26)	<0.001	4

AMI, acute myocardial infarction; eGFR, estimated glomerular filtration rate; No., number; PCI, percutaneous coronary intervention; SES, sirolimus-eluting stent. VLST, very late stent thrombosis.

### Risk-score validation

The c-statistic of the final model was 0.83 (95% CI: 0.78–0.89) in the validation cohort, with a p-value of 0.66 from the Hosmer-Lemeshow test. The c-statistic of the VLST risk score was 0.82 (95% CI: 0.76–0.90), with a p-value of 0.97 from the Hosmer-Lemeshow test. The HR of the risk score was 1.74 for the validation cohort (95% CI: 1.53–1.99). The predicted and observed incidences of VLST across deciles of the risk score in the validation cohort are illustrated in Supplementary Fig. S2. According to predicted risk, 64.67% (n = 1,331), 26.97% (n = 555), and 8.36% (n = 172) of the validation cohort were identified as low, intermediate, and high risk, respectively. The observed incidences of VLST in each risk group were 0.45%, 2.34%, and 9.3%, respectively, in the validation cohort (Fig. 1). The risk scores and the occurrence of VLST in the derivation and validation cohorts are depicted in Supplementary Fig. S3.

**Figure 2:**
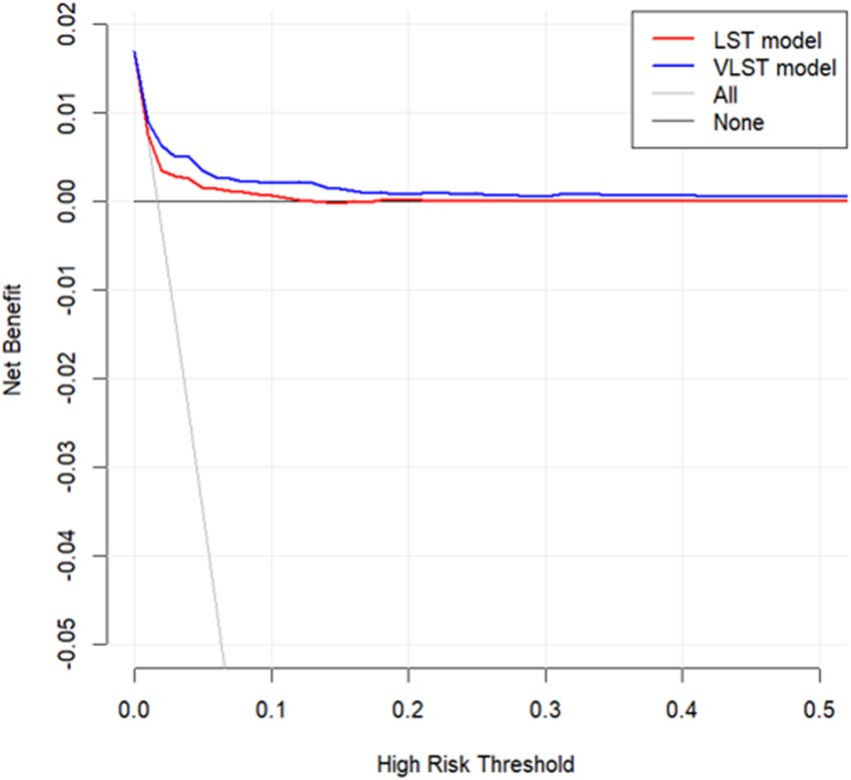
Decision-curve analysis. Net benefit of using a model to predict VLST compared with strategies of ‘assuming all’ or ‘assuming none’ patients would be at high-risk for different decision thresholds is shown. The VLST risk score (blue) shows improved benefit compared with the LST risk score (red). LST, late stent thrombosis; VLST, very late stent thrombosis.

The prediction model was further evaluated by decision-curve analysis (Fig. 2). The results demonstrated that, for relevant decision thresholds, the VLST risk-score model provided a substantial net clinical benefit compared with that of the LST risk-score model, which included only three variables (current smoking, history of PCI, and ST-elevated MI [STEMI] as the admitting diagnosis). For a decision threshold of 2% of VLST risk, compared with the LS risk score, the VLST risk score should identify 4 additional cases, without any false-positive results, from a population of 1,000 patients with a 1.7% incidence of VLST.

## Discussion

**Figure 3:**
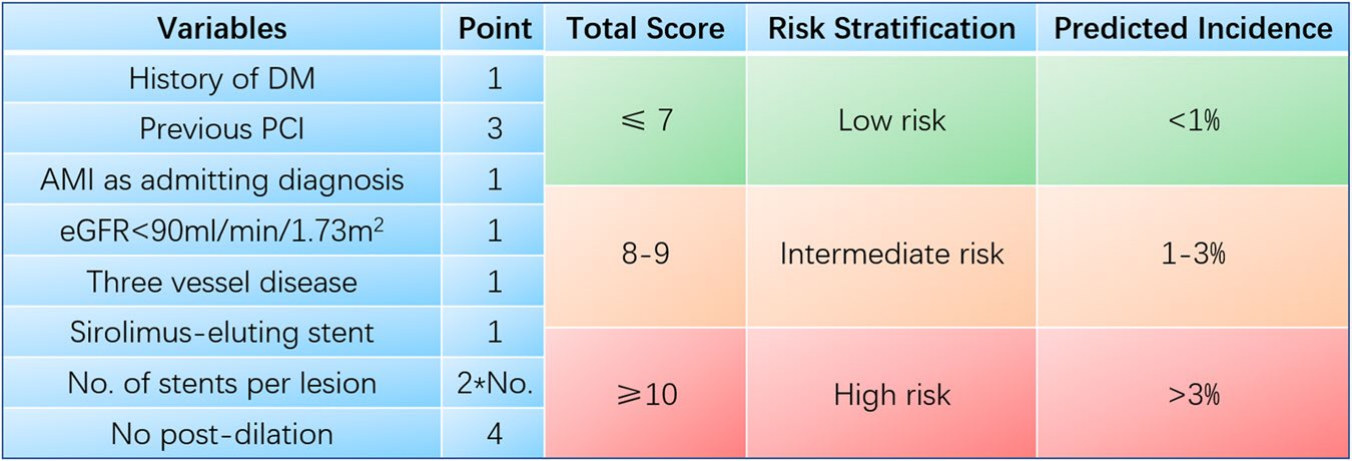
The VLST scoring system. AMI, acute myocardial infarction; DM, diabetes mellitus; eGFR, estimated glomerular filtration rate; PCI, percutaneous coronary intervention; VLST, very late stent thrombosis.

In this study, development of VLST was independently associated with the following eight variables: history of DM, previous PCI, acute myocardial infarction as admitting diagnosis, estimated glomerular filtration rate (eGFR)<90 ml/min/1.73 m^2^, three-vessel disease, number of stents per lesion, SES, and no post-dilation. The observed VLST rates were 0.5%, 2.2%, and 8.7% in the derivation cohort and 0.45%, 2.3%, and 9.3% in the validation cohort across the low-, intermediate-, and high-risk categories, respectively. High discrimination (c-statistic of 0.80 and 0.82 in the derivation and validation cohorts, respectively) and excellent calibration were noted in both cohorts. To the best of our knowledge, this is the first study to provide a simple but useable scoring system for identifying impending VLST after PCI from a large cohort of consecutive patients with ACS. The VLST risk score (  Fig. 3) showed good predictive ability and calibration across the two cohorts. Being based on simple categorical variables, the score could be used to identify high-risk patients, with particular utility in the decision of treatment plans for patients more than 1 year after PCI.

Previous clinical studies have divided the factors that are correlated with the development of VLST into three categories: patient- and lesion-related factors^4–6^, procedural- and stent-related factors^7–11^, and pharmacotherapy-related factors^12,13^. In the present study, five variables were patient- and lesion-related and three were procedural- or stent-related, consistent with previous literature reports^4–13^. The effect of discontinuation of DAPT, which has been suggested to affect the development of VLST during follow-up^14^, had no statistically significant effect on the incidence of VLST in the derivation cohort.

Patient- and lesion-related variables included a history of DM, previous PCI, acute MI as the admitting diagnosis, eGFR <90 ml/min/1.73 m^2^, and three-vessel disease. Previous studies have suggested that DM plays an important role in promoting atherosclerosis, and in-stent neoatherosclerosis has been found to be an important underlying mechanism of VLST^15,16^. Additionally, a history of DM has been described as a powerful predictor of subacute, late, and very late stent thrombosis^17^. Previous PCI might indicate prior stent placement and has been included as a variable in the LST risk score^3^. Previous studies have reported higher rates of stent thrombosis (ST) among patients with a history of MI, which might be related to the different plaque characteristics underlying the MI and angina pectoris^18–20^. Chronic kidney disease could promote the progression of arteriosclerosis and has been found to be an independent risk factor for ST^21,22^. The three-vessel disease is an indicator of the severity and diffusion of lesions and is correlated with the development of ST^6^.

Regarding the procedure- and stent-related variables, a greater number of stents indicates longer lesions and stents; thus, more stent overlap, all of which could contribute to the development of ST^6^. Furthermore, no post-dilation was reported as an independent risk factor for VLST in the case of bioresorbable stents^23^, and might lead to stent malposition, which is an important mechanism underlying VLST development^7,24,25^. The representativeantiproliferation-drug-eluting stents of the first-generation drug-eluting stents (DESs) are SESs; local toxicity and inflammatory reactions after implantation of these stents could lead to delayed endothelialisation or even positive remodelling of the vessel walls. This is also an important mechanism involved in the development of VLST^16,26–30^.

In clinical practice, VLST often occurs more than 1 year after the initial stent placement; therefore, identifying patients at high risk for VLST is extremely important as it may enable the implementation of intensive follow-up and preventative interventions. The LST risk score was established in a cohort of selected patients with ACS and has proven to be equally useful in predicting the occurrence of VLST. The system includes three variables as follows: current smoker, history of previous PCI, and STEMI as the admission diagnosis, but the predictive ability of the score is limited (c-statistic = 0.66)^3^. A recent study^31^ presents a new risk score developed from a small sample case-control study; however, no validation data were reported for this system, and some variables, such as suboptimal stent result, that were included in the score cannot be assessed objectively. Compared with the above study^3,31^, the strength of our study is the inclusion of a cohort of consecutive patients with ACS, which can be expected to reflect real-world clinical practice, and improved discrimination was shown in both the derivation and validation cohorts.

The VLST risk score provides a simple but efficient tool to identify patients at high risk for VLST after PCI. Intensive follow-up should be carried out for these patients, and more aggressive therapy might be required. The independent risk factors included in the present risk model will be significant in guiding the choice of therapeutic approach. For example, selecting fewer stents, using new-generation DESs, and performing effective post-dilation could reduce the incidence of ST. However, the fact that the VLST risk score presented here was developed and validated in patients with ACS means that its value in patients with stable angina pectoris still needs to be proven.

Part of the inclusion criteria for this study was definite ST (assessed by coronary angiography [CAG]) to ensure consistency of events. This might have resulted in some events being missed, though we tried to reduce this situation. In addition, although a single-centre cohort was used for validation, the reliability of the score warrants validation using multi-centre, large-scale prospective cohorts.

The VLST risk score presented here provides a readily useable and efficient tool to identify patients with ACS who are at high risk of developing VLST after PCI. The system may enable further risk stratification and thus, facilitate pre-emptive clinical decision-making.

## Methods

### Study population

This study is registered at ClinicalTrials.gov: NCT03491891. We recruited consecutive patients ≥18 years who were admitted to The First Hospital of Jilin University between January 1, 2014 and June 1, 2015 and underwent PCI for treatment of ACS and who had signed informed consent. The exclusion criteria were as follows: expected survival time of less than 1 year, severe organ failure, malignancy, a surgical plan for important organs within 1 year after PCI and antiplatelet drugs should be discontinued; death prior to discharge; and refusal to sign informed consent or take part in follow-up interviews or patients who were lost to follow-up after discharge.

For the external validation cohort, we recruited all consecutive ACS patients who were admitted to the first affiliated hospital of Shantou University for PCI between January 1, 2014 and June 1, 2015, and the same exclusion criteria were applied.

All patients enrolled were treated with newer or second-generation DESs (Supplementary Table S3) and DAPT, lasting for at least one year. Antiplatelet therapy continued beyond this time was based on the decision of patients’ personal doctors considering their specific conditions (Supplementary Table S4).

In order to improve the diagnosis of VLST, we set up a 24-hour online consultation to ensure the patients enrolled would receive professional medical evaluation and medical advice immediately when they have cardiac symptoms. For highly suspicious patients, they will be immediately transferred to the nearby Chest Pain Center, and CAG images of confirmed patients will be uploaded to the core laboratory for analysis.

### Ethical information

Ethics committee approval (NO. 2013-256) was obtained from the appropriate committee of both institutes (Medical Ethics Committee of The First Hospital of Jilin University, First Affiliated Hospital of Shantou University Medical College Ethics Committee), and the study was performed in accordance with the ethical standards laid out in the 1964 Declaration of Helsinki and its later amendments. All patients provided written informed consent.

### Definitions and endpoints

We defined VLST according to the 2007 criteria of the Academic Research Consortium (ARC) for ST; specifically, ST that occurred more than one year after coronary stent implantation and was confirmed by CAG. All documented cases of VLST were determined by CAG, and the procedures were independently reviewed by two experienced interventional cardiologists. If a consensus could not be reached between the two reviewers, a third reviewer was consulted. The primary endpoint of this study was definite ST.

### Statistical analysis

Categorical data are presented as numbers (percentage). Continuous data are presented as mean ± standard deviation. Categorical data were compared using the chi-square test or Fisher’s exact test, while quantitative data were compared using the Mann-Whitney U test. All statistical tests were 2-sided with a 0.05 significance level.

The derivation cohort was used to identify predictors for the occurrence of VLST and to establish a risk-scoring system, which was verified using the validation cohort. Significant variables (P <0.05) in univariable Cox regression analysis and independent risk factors reported in previous literature were included in the multivariate Cox regression analysis and the optimal predictive variables of VLST were identified using the backward stepwise method. Some variables were excluded, such as leukocyte count (for which there was insufficient evidence to definitively conclude that the increased count was not owing to infection or other factors). Procedural variables, such as stent diameter, length, type, and post-dilation, were based on the operator’s subjective assessment during PCI.

Multicollinearity between the variables in the final model was assessed by calculation of the variance-inflation factor. These corrected coefficients of significant predictors were then divided by the value of the lowest coefficient and rounded to the nearest integer to assign a weight to each predictor. The total score was calculated for each patient by adding these weights together. An objective assessment of calibration was obtained using the Hosmer-Lemeshow goodness-of-fit test and by plotting the observed vs. predicted incidence rates across the risk-score deciles. The predictive performance of the ris model was assessed using the c-statistic and was cross-validated (using the leave-one-out method) in the development cohort. Clinical utility was evaluated using decision-curve analysis. Statistical analyses were performed using the Statistical Package for Social Sciences version 23 (SPSS, Chicago, IL, USA) and R version 3.5.0 (R Foundation for Statistical Computing, Vienna, Austria) with the rms package.

## Supplementary information

Supplementary Information.

## Data Availability

All access data are available at www.figshare.com.
