# Supplementary material for: A scoring system to predict the occurrence of very late stent thrombosis following percutaneous coronary intervention for acute coronary syndrome
Source: Sci Rep. 2020 Apr 14;10:6378. doi: 10.1038/s41598-020-63455-0 (PMC7156476; doi:10.1038/s41598-020-63455-0)
Supplement: Supplementary file 1 — Supplementary Information. [file 41598_2020_63455_MOESM1_ESM.pdf]

Supplemental Material

A scoring system to predict the occurrence of very late stent thrombosis following percutaneous coronary intervention for acute coronary syndrome

Xiang Wang[1,a], PhD; Xinxin Chen[1,a], PhD; Tao Tian[1], MD; Hongzhao You[2], MD; Yulin Li[3], PhD; Muli Wu[4], MD; Xiaoyu Du[1], PhD; He Cai[1], MD; Yang Zheng[1*], PhD; Jie Du[3*], PhD

1. **Supplementary tables**

Supplementary Table S1. Baseline, procedural characteristics, and laboratory test results in the derivation and validation cohorts

Variables	Derivation cohort (n = 5185)	Validation Cohort (n = 2058)	p value
Age (years)	59.846 ± 9.95	60.00 ± 10.22	0.248
Male sex	3557 (68.60)	1414 (68.71)	0.930
Diabetes Mellitus	1329 (25.63)	550 (26.73)	0.338
Hypertension	2721 (52.48)	1073 (52.14)	0.794
Dyslipidaemia	1640 (31.63)	515 (25.02)	< 0.001
Current smoker	2914 (56.20)	1202 (58.41)	0.087
History of drinking	764 (14.74)	327 (15.89)	0.215
History of stroke/TIA	379 (7.31)	170 (8.26)	0.168
History of PVD	88 (1.70)	42 (2.04)	0.321
History of heart failure	137 (2.64)	58 (2.82)	0.676
Previous CABG	144 (2.78)	65 (3.16)	0.382
Previous PCI	104 (2.01)	46 (2.24)	0.536
Previous MI	357 (6.89)	114 (5.54)	0.036
AMI as admitting diagnosis	3160 (60.95)	1258 (61.13)	0.886
Cardiogenic shock	113 (2.18)	17 (0.83)	< 0.001
Three vessel disease	1464 (28.24)	575 (27.94)	0.801
Chronic total occlusion	239 (4.61)	52 (2.53)	< 0.001
Moderate to severe calcification	185 (3.57)	50 (2.43)	0.014

Moderate to severe tortuosity	60 (1.16)	18 (0.88)	0.293
Ostial lesion	119 (2.30)	69 (3.35)	0.011
Proximal lesion	3544 (68.35)	1576 (76.58)	< 0.001
Bifurcation lesion	638 (12.31)	104 (5.05)	< 0.001
Visual thrombus	598 (11.53)	187 (9.08)	0.003
Coronary aneurysm	28 (0.54)	12 (0.58)	0.823
Vessel dilation	59 (1.14)	29 (1.41)	0.342
Vessel ulceration	39 (0.75)	25 (1.22)	0.058
Vessel dissection	53 (1.02)	41 (1.99)	< 0.001
Culprit vessel			
LM	105 (2.03)	26 (1.26)	0.028
LAD	2729 (52.63)	1035 (50.29)	0.072
LCX	896 (17.28)	358 (17.40)	0.907
RCA	1721 (33.19)	714 (34.69)	0.222
TIMI flow grade 0 before PCI	1709 (32.96)	688 (33.43)	0.701
Thrombus aspiration	664 (12.81)	361 (17.54)	< 0.001
Slow flow after PCI	188 (3.63)	47 (2.28)	0.004
No reflow after PCI	44 (0.85)	28 (1.36)	0.048
Stent type used during PCI			
SES	3578 (69.01)	1429 (69.44)	0.721
ZES	898 (17.32)	343 (16.67)	0.506
EVS	709 (13.67)	286 (13.90)	0.762

Stent overlap	995（19.19）	422（20.51）	0.203
No post-dilation	2510（48.41）	1124（54.62）	< 0.001
Reference vessel diameter (mm)	3.02 ± 0.40	3.02 ± 0.39	0.642
Min-stent diameter (mm)	2.99 ± 0.41	2.99 ± 0.39	0.554
Max-stent diameter (mm)	3.06 ± 0.41	3.06 ± 0.40	0.702
Total stent length (mm)	31.82 ± 15.75	32.20 ± 16.14	0.434
Stent release pressure (atm)	13.95 ± 2.98	13.84 ± 4.54	0.011
No. of stents per lesion	1.217 ± 0.46	1.23 ± 0.48	0.261
LVEF	55.14 ± 4.50	55.52 ± 5.83	< 0.001
eGFR < 90 (ml/min/1.73m^2)	892（17.20）	335（16.28）	0.344
WBC (*10^9/l)	8.81 ± 3.29	8.67 ± 3.30	0.038
HGB (g/l)	141.69 ± 16.17	141.89 ± 16.19	0.656
Platelet (*10^9/l)	225.29 ± 62.38	225.86 ± 61.45	0.685
TCL (mmol/l)	4.57 ± 1.05	4.67 ± 1.09	< 0.001
LDL (mmol/l)	2.86 ± 0.82	2.84 ± 0.80	0.346
HDL (mmol/l)	1.13 ± 0.28	1.17 ± 0.30	< 0.001
TG (mmol/l)	2.12 ± 1.40	2.19 ± 1.42	0.047
Fasting blood glucose (mmol/l)	6.72 ± 2.76	6.58 ± 2.84	< 0.001
HbA1c (%)	5.81 ± 1.61	6.03 ± 1.65	< 0.001
Fibrinogen (g/l)	3.17 ± 0.88	3.36 ± 0.91	< 0.001
DAPT	2295 (44.26)	900 (43.73)	0.682

*Values are the number (%) or mean±SD. p-values represent differences between groups.

* AMI, acute myocardial infarction; CABG, coronary artery bypass graft; EES, everolimus eluting stent; HDL high density lipoprotein;HGB, hemoglobin; LM, left main; LAD, left anterior descending artery; LCX, left circumflex artery; LDL, low density lipoprotein; LVEF, left ventricular eject fraction; MI, myocardial infarction; PVD, peripheral vascular disease; PCI, percutaneous coronary intervention; RCA, right

coronary artery; SES, sirolimus eluting stent; TIMI, Thrombolysis In Myocardial Infarction; TCL, total cholesterol; TG, triglyceride. TIA, transient ischemic attack; WBC, white blood cell; ZES, zotarolimus eluting stent.

Supplementary Table S2. Variables associated with the occurrence of VLST in univariable analysis in the derivation cohort and independent risk factors of VLST reported in literature

Variables	VLST (n = 92)	No VLST (n = 5093)	HR (95%CI)	p value
Diabetes Mellitus	36	1293	1.88(1.24-2.86)	0.003
Current smoker	54	2860	1.10(0.73-1.67)	0.642
Stroke/TIA	12	367	1.98(1.08-3.64)	0.027
Previous PCI	13	94	5.88(3.04-11.34)	< 0.001
AMI as admitting diagnosis	72	3046	1.56(0.99-2.44)	0.053
Three vessel disease	42	1422	2.17(1.44-3.27)	< 0.001
Bifurcation lesion	17	621	1.67(0.99-2.83)	0.056
TIMI flow grade 0 before PCI	41	1668	1.64(1.08-2.47)	0.019
Stent type-SES	76	3502	2.10(1.23-3.60)	0.007
Stent overlap	27	968	1.76(1.12-2.75)	0.014
No post-dilation	78	2597	0.20(0.11-0.35)	< 0.001
Total stent length	38.46 ± 20.71	31.70 ± 15.62	1.02（1.01-1.03）	< 0.001
No. of stents per lesion	1.42 ± 0.65	1.21 ± 0.46	1.99(1.45-2.73)	< 0.001
LVEF	54.55 ± 3.68	55.15 ± 4.52	0.98(0.936-1.02)	0.225
eGFR < 90(ml/min/1.73m2)	32	860	2.59(1.68-3.97)	< 0.001
Fibrinogen (g/l)	3.37 ± 1.01	3.17 ± 0.88	2.42(1.14-5.16)	0.022

* AMI, acute myocardial infarction; eGFR, estimated glomerular filtration rate; PCI, percutaneous coronary intervention; TIA, transient ischemic attack; TIMI, thrombolysis in myocardial infarction; LVEF, left ventricular eject fraction; SES, sirolimus eluting stent; VLST, very late stent thrombosis.

Supplementary Table S3. Parameters of the stents used in the study

Stent Brand	Polymer	Biocompatibility	Thickness of polymer(μm)	Drug	Strut thickness (μm)
Partner	PBMA/PEVA	durable	6	Sirolimus	100
Excel	PLA	biodegradable	10-15	Sirolimus	120
Tivoli	PLGA	biodegradable	5.5	Sirolimus	80
Firebird2	SBS	durable	6.0	Sirolimus	86
Endeavor Resolute	BioLinx	biocompatible	5.6	Zotarolimus	91
Promus Element	PBMA,PVDF-HFP	durable	7.0	Everolimus	81
XienceV	PBMA,PVDF-HFP	durable	7.6	Everolimus	81
Xience Xpedition	PBMA,PVDF-HFP	durable	7.6	Everolimus	81
Xience Prime	PBMA,PVDF-HFP	durable	7.6	Everolimus	81

*PBMA, poly butyl methacrylate; PEVA, poly ethyleneco vinyl acetate; PLA, polylactic acid; PLGA, polylactide-co-glycolide; SBS, styrene-butadiene block copolymer; BioLinx™,blend of a hydrophobic C10 polymer, a hydrophilic C19 polymer, and a hydrophilic polyvinyl-pyrrolidinone; PVDF-HFP, Fluoropolymer, consists of acrylic and fluoro polymers; Co-Cr, cobalt-chromium; Pl-Cr, platinum-chromium.

Supplementary Table S4. Dual antiplatelet therapy in the derivation and validation cohort at the end of follow-up

Patients	With DAPT	Without DAPT
Derivation Cohort-with VLST	35	57
Derivation Cohort-without VLST	2260	2833
Validation Cohort-with VLST	11	24
Validation Cohort-without VLST	889	1134

* DAPT, Dual antiplatelet therapy; VLST, very late stent thrombosis.

2. Supplementary figures

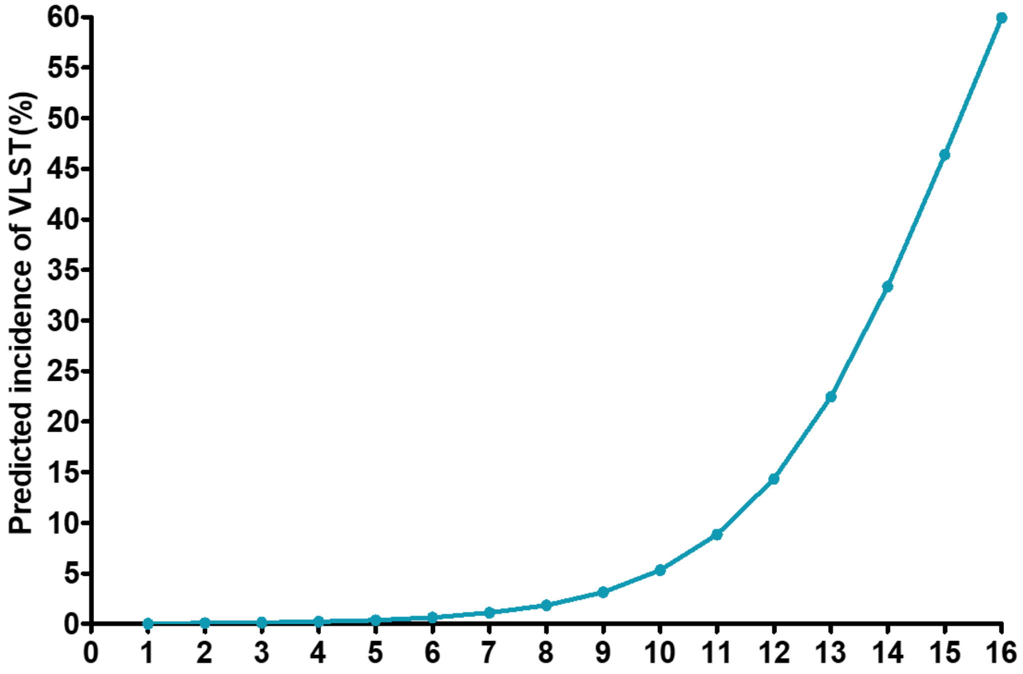

Supplementary Fig. S1 Relationship between the score value and the predicted incidence of VLST in the derivation cohort.

*VLST, very late stent thrombosis.

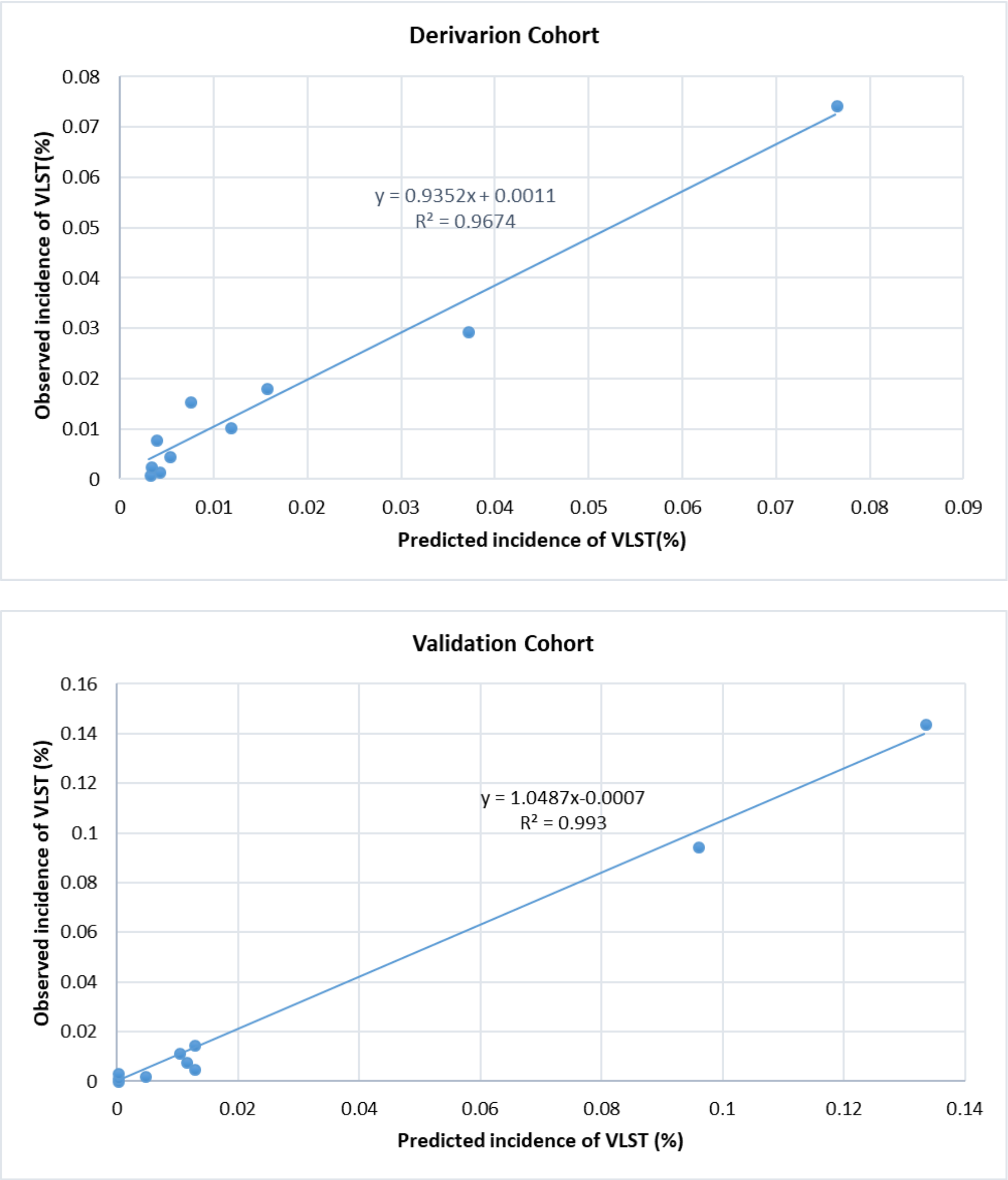

Supplementary Fig. S2 Calibration plots. Calibration plots showing the predicted probability vs. observed incidence of VLST in the derivation and validation cohorts.

*VLST, very late stent thrombosis.

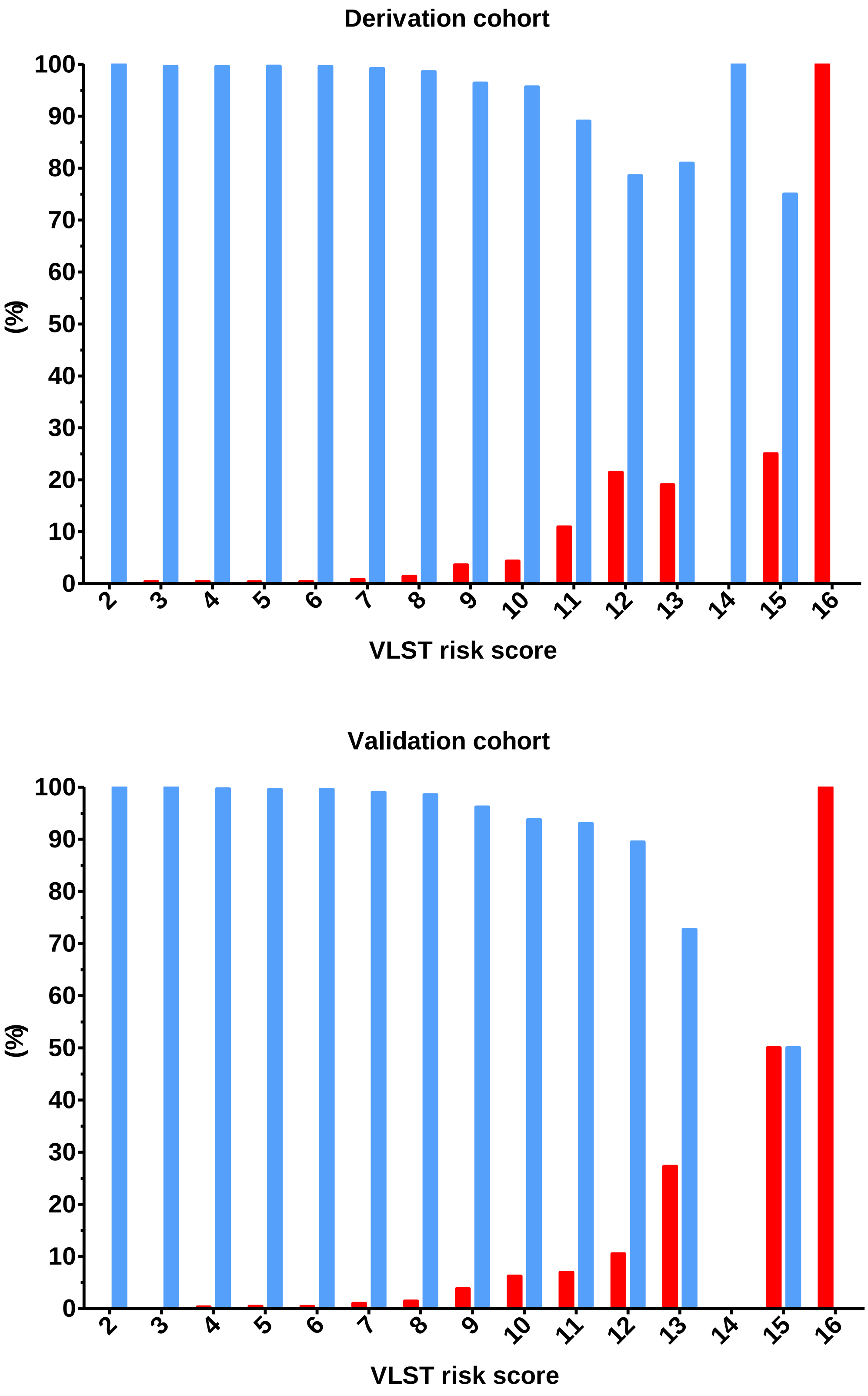

Supplementary Fig. S3 Distributions of patients according to risk score and the occurrence of VLST in the derivation and validation cohorts.

*VLST, very late stent thrombosis.
